# Stromal cells support the survival of human primary chronic lymphocytic leukemia (CLL) cells through Lyn-driven extracellular vesicles

**DOI:** 10.3389/fmed.2022.1059028

**Published:** 2023-01-13

**Authors:** Thaís Dolzany de Oliveira, Alexander vom Stein, Rocio Rebollido-Rios, Liudmila Lobastova, Marcus Lettau, Ottmar Janssen, Prerana Wagle, Phuong-Hien Nguyen, Michael Hallek, Hinrich P. Hansen

**Affiliations:** ^1^Department I of Internal Medicine, Center for Integrated Oncology Aachen Bonn Cologne Düsseldorf, University of Cologne, Cologne, Germany; ^2^Center for Molecular Medicine Cologne, University of Cologne, Cologne, Germany; ^3^CECAD Center of Excellence on Cellular Stress Responses in Aging-Associated Diseases, University of Cologne, Cologne, Germany; ^4^Institute of Immunology, University Hospital Schleswig-Holstein, Christian-Albrecht University of Kiel, Kiel, Germany; ^5^Department of Hematology, University Hospital Schleswig-Holstein, Kiel, Germany; ^6^CECAD Center of Excellence on Cellular Stress Responses in Aging-Associated Diseases, Proteomics Facility, University of Cologne, Cologne, Germany

**Keywords:** chronic lymphocytic leukemia, extracellular vesicle, Lyn kinase, extracellular matrix, CD248, filopodia

## Abstract

**Introduction:**

In chronic lymphocytic leukemia (CLL), the tumor cells receive survival support from stromal cells through direct cell contact, soluble factors and extracellular vesicles (EVs). The protein tyrosine kinase Lyn is aberrantly expressed in the malignant and stromal cells in CLL tissue. We studied the role of Lyn in the EV-based communication and tumor support.

**Methods:**

We compared the Lyn-dependent EV release, uptake and functionality using Lyn-proficient (wild-type) and -deficient stromal cells and primary CLL cells.

**Results:**

Lyn-proficient cells caused a significantly higher EV release and EV uptake as compared to Lyn-deficient cells and also conferred stronger support of primary CLL cells. Proteomic comparison of the EVs from Lyn-proficient and -deficient stromal cells revealed 70 significantly differentially expressed proteins. Gene ontology studies categorized many of which to organization of the extracellular matrix, such as collagen, fibronectin, fibrillin, Lysyl oxidase like 2, integrins and endosialin (CD248). In terms of function, a knockdown of CD248 in Lyn^+^ HS-5 cells resulted in a diminished B-CLL cell feeding capacity compared to wildtype or scrambled control cells. CD248 is a marker of certain tumors and cancer-associated fibroblast (CAF) and crosslinks fibronectin and collagen in a membrane-associated context.

**Conclusion:**

Our data provide preclinical evidence that the tyrosine kinase Lyn crucially influences the EV-based communication between stromal and primary B-CLL cells by raising EV release and altering the concentration of functional molecules of the extracellular matrix.

## Introduction

Malignant B cells in chronic lymphocytic leukemia (CLL) undergo rapid apoptosis when isolated from peripheral blood of patients (1). *In situ*, however, they are protected from apoptosis by a plethora of coordinated interactions with typically activated supporter cells and biological products they release. In CLL patients, the secondary lymphoid organs provide a characteristic tumor microenvironment (TME) where the malignant cells receive this pivotal survival support. It contains not only supporter cells but also supporting structures within the extracellular matrix (2). Among the supporter cells, mesenchymal stromal cells and follicular dendritic cells play an important role. They interact with CLL cells not only through direct cell contact and soluble factors but also through released extracellular vesicles (EV). EVs are double membrane-enclosed small particles that transport lipids, nucleic acids, and proteins (3). They are released by exocytosis of multivesicular endosomes as small EVs (s-EVs, 40–120 nm) or by budding from the plasma membrane as large EVs (l-EVs, 100–1,000 nm). Both EV types are functional, carry typical traits of the donor cells and form a novel avenue of intercellular communication. In addition to the payload from the donor cell, they also carry molecules that specifically bind to the EVs. This corona also participates in their functionality (4). EVs from stromal cells are taken up by CLL cells and stimulate their migration and survival (5). On the other hand, EVs from CLL cells induce the transition of stromal cells to CLL-supporting cancer-associated fibroblasts (CAF) (6). It becomes increasingly clear that EVs decisively participate in the mutual stimulation of tumor and bystander cells.

The infiltration of stromal cells in the TME of CLL tissue is an essential contribution to the survival of primary CLL cells. CLL cells are able to differentiate stromal cells to CAFs, which in turn directly support CLL cells and cause angiogenesis by stimulation of endothelial cells (7). Stromal cells also release fibrogenic factors, such as fibroblast growth factor (FGF), transforming growth factor beta1 (TGFB1), collagen, and lysyl oxidase (LOX), the latter enzyme increases the stiffness of the matrix by crosslinking collagen fibers and elastin (8). Another important molecule in this context is the transmembrane protein CD248 (endosialin, tumor endothelial marker 1/TEM1). It is selectively expressed on the surface of CAFs and pericytes and released in EVs (9). CD248 crosslinks collagen and fibronectin fibers and might contribute to the stiffness of the extracellular matrix (ECM) (10). Activated Lyn can induce ECM stiffness by activating the twist 1 pathway (11). A dense collagenous stroma is a strong indicator of tumor aggressiveness and therapeutic resistance (12).

Recent data suggest that EV-based communication is regulated. In addition to a certain constitutive production of EVs, their release can be stimulated by certain conditions of the TME, such as hypoxia, extracellular ATP, STEAP3, or elevated Ca^2+^ (13). CLL cells release more EVs after B cell receptor (BCR) stimulation and less upon Bruton tyrosine kinase (BTK) inhibition, suggesting that kinases are involved in the regulation of the EV release (14). Lyn, a Src family kinase (SFK), is expressed in tumor and bystander cells of CLL affected tissue and correlates with progression of the disease (15). Lyn is also activated by BCR stimulation and then phosphorylates the BCR adaptors CD79a/b to stimulate a survival signaling cascade involving the kinases BTK and PI3K. It also causes actin nucleation through stimulation of the cytoskeleton activator HS1 (hematopoietic cell-specific Lyn substate 1) (16, 17). The latter results in the formation of protrusions, filopodia, and membrane blebs. In primary CLL cells with an active Lyn-HS1 axis, the treatment with the Lyn inhibitor Dasatinib results in a reduction of the Lyn activity, HS1 phosphorylation and F-actin polymerization (18). All in all, the contribution of the Lyn kinase to EV formation and release from stromal cells and EV-based communication with CLL cells is still far from being completely understood. To shed light on a potential interdependency between stromal and CLL cells, we compared Lyn-proficient and deficient stroma cell lines to study the influence of the kinase on the EV-based, tumor-supporting communication.

## Materials and methods

### Cells and reagents

The cell lines StromaNKtert (CVCL_4667), HS-5 (CRL-11882), WI-38 (ATCC#CCL-75), MEC-1 (CVCL_1870) were cultivated at 37°C and 5% CO_2_ in RPMI 1640 containing 10% FBS, supplemented with GlutaMAX (2 mM), 100 U/ml penicillin and 100 μg/ml streptomycin. The following additional reagents were used: PE annexin V (Cat# 640907, BioLegend), rabbit anti-human CD248 (AB67273, Abcam, Berlin, Germany), anti-β-actin antibody (Santa Cruz), Vybrant-DiD (red, 40 nM 1,1’-Dioctadecyl-3,3,3’,3’-tetramethylindo-dicarbo-cyanine, 4-chlorobenzenesulfonate salt, Thermo Fisher Scientific, Munich, Germany), CFSE (green, 6-carboxyfluorescein succinimidyl ester, Thermo Fisher Scientific, Munich, Germany), CellTiter-Glo 2.0 (Promega, Walldorf, Germany).

### Isolation of primary CLL cells

Blood of CLL patients was obtained from the CLL-Biobank of the University Hospital of Cologne, after written informed consent. B-CLL cells were isolated from the whole blood using the RosetteSep™ Enrichment Cocktail and incubated for 20 min. The samples were diluted with an equal volume of phosphate-buffered saline (PBS) + 10% fetal bovine serum (FBS), mixed gently and subsequently added on top of the density medium. This gradient was centrifuged for 20 min at 1,200 × *g* at room temperature, with the brake switched off. The enriched B-CLL cells were collected from the interphase and washed twice with PBS + 10% FBS. The harvested cells were maintained in RPMI-1640 medium with 10% FBS for direct use or frozen with 10% dimethyl sulfoxide (DMSO) solution.

### Vesicle isolation

HS-5 cells were cultivated under serum-free conditions (StromaNKtert with 5% vesicle-depleted serum) at 5 × 10^6^/ml for 16 h. The cell supernatant was collected and cleared by three consecutive centrifugation steps, i.e., 5 min at 300 × *g*, 10 min at 3,000 × *g*, 20 min at 10,000 × *g*. Cleared supernatants were sedimented in an ultracentrifuge for 90 min at 110,000 × *g*. The EV pellet was suspended in 500 μL PBS and purified on qEVoriginal 35 nm (IZON Science, Lyon, France). The EV-containing fractions were controlled by nanoparticle tracking analysis (NTA, Nanosight NS300, Malvern Instruments, Malvern, UK) and the protein concentration determined by NanoDrop and bicinchoninic acid (BCA) protein assay.

### Extracellular vesicle uptake analysis

Extracellular vesicles were isolated and washed with PBS by ultracentrifugation for 90 min at 110,000 × *g*. Then, they were labeled with Vybrant-DiD or CFSE for 20 min at 37°C. EVs were washed twice with PBS by ultracentrifugation at 110,000 × *g* for 90 min. Stained EVs were incubated with target cells for 24 h. Then, cells were washed with PBS and analyzed by flow cytometry. For imaging flow cytometry, cells were fixed with 1% paraformaldehyde for 20 min on ice and washed and suspended in FACS buffer.

### Determination of EV release

Cells (2 × 10^6^/ml) were cultivated for 24 h in a six-well plate to allow adherence. They were washed 3 times with PBS and further incubated for 3 h in serum-free RPMI-1640. Then, the supernatant was collected, the EVs were purified and analyzed by Nanoparticle Tracking Analysis.

### Co-culture of primary cells with EVs

Primary B-CLL cells (0.3 × 10^6^ cells/well) were cultured in RPMI-1640/10% FBS with or without 4 μg/ml of EVs in 96-well plates. Cell viability was determined by CellTiter-Glo 2.0, according to the manufacturer’s protocol.

### Co-culture of primary cells with stromal cells

Primary B-CLL cells (1.5 × 10^6^/well in 24-well plate) were cultured in RPMI-1640/10% FBS with or without HS-5 wt, HS-5 scr, Lyn-deficient or CD248-deficient HS-5 (1 × 10^4^/well) cells. The floating CLL cells were removed after different incubation periods (24, 48, 72, and 96 h) and transferred to a new plate where the viability was determined by CellTiter-Glo 2.0.

### Cryo-transmission electron microscopy

The vesicle pellet was suspended in 50 μL PBS. Approximately 3 μL were applied on a copper grid (Quantifoil S7/2 Cu 400 mesh, carbon films; Quantifoil Micro Tools GmbH, Jena, Germany). After removal of excessive liquid, the grids were immediately shock-frozen by injection into liquid ethane. The grids were transferred into the transmission electron microscope (Leo 912 Ω-mega) and analyzed at −174°C. The instrument was operated at 120 kV and pictures with a 6,300- to 12,500-fold magnification were taken.

### Confocal laser-scanning microscopy

Cells were let to adhere in a six-well plate prepared with coverslips and incubated with the target EVs (stained with CFSE) for 3 or 24 h. Next, the cells were fixed with paraformaldehyde 4% and permeabilized with Triton X-100 0.01% for 15 min, followed by washing. Then, the medium was carefully removed and replaced with fresh medium, containing CellMask™ Deep Red Plasma membrane stain (649/666 nm; Thermo Fisher Scientific, Munich, Germany) and Hoechst 33342 dye (NucBlue™ Live ReadyProbes™, Thermo Fisher Scientific, Munich, Germany). After 20 min of incubation, the coverslips were washed and mounted onto the slides with a mounting medium, and finally the samples were subjected to the confocal laser scanning microscope (Leica TCS SP8, 63× PlanApo oil objective N.A. 1.4) with super resolution (∼50 nm lateral, 120 nm axial). The images were analyzed using Fiji software.

### Measurement of EV uptake by imaging flow cytometry (ImageStream)

Lyn-proficient or Lyn-deficient HS-5 cells were cultivated with purified DiD-labeled EVs from MEC-1 CLL cells for 24 h. Then, cells were washed once with cold FACS-buffer, fixed with 1% paraformaldehyde (PFA) in PBS and subsequently kept on ice until internalization was quantified by imaging flow cytometry using an ImageStream X Mark II one camera system with 351, 488, 562, 658, and 732 nm lasers (Merck Millipore, Burlington, MA, USA). The system was calibrated using SpeedBeads (Merck Millipore, Burlington, MA, USA) prior to use and at least 10,000 events were acquired. Moreover, 500–1,000 events of single stained compensation control samples gated on appropriate signal size were acquired with both the bright field channel and the 732 nm laser turned off. Images [bright field in channel 1 and fluorescein-5-isothiocyanate (FITC) in channel 2 (505–560 nm)] were acquired at 60-fold magnification. The integrated software INSPIRE^®^ was used for data collection as raw image files. Single color controls were used to calculate a spectral crosstalk matrix that was applied to each raw image file for spectral compensations in the detection channels. The analysis was performed on the compensated image files using the IDEAS^®^ image analysis software. The bright field gradient root mean square (RMS) feature was used to gate on focused cells and dot plots of the bright field area versus the aspect ratio were used to gate on single cells. The internalization wizard was used to calculate the internalization score that is defined as the ratio of the intensity inside the cell to the intensity of the entire cell mapped to a logarithmic scale.

### Filopodia analysis with confocal microscopy

HS-5 cells were seeded (0.3 × 10^6^ cells/ml) in a six-well plate with a coverslip and cultivated for 24 h. The cells were then fixed with paraformaldehyde 4% for 15 min, washed with PBS, and permeabilized with Triton X-100 (0.01%) for 15 min, followed by PBS washing and staining of the nuclei and actin for 20 min. The images were acquired using a SP8 Leica confocal microscope with 63× magnification and analyzed using Fiji software. According to the original protocol, the Fiji macro FiloQuant plugin was used to measure the filopodia number and length (19).

### Generation of Lyn-deficient stromal cells

HS-5 cells: Lyn-deficient HS-5 cells were generated using a CRISPR-Lyn knockout kit (Origene, Rockville, USA). Stromal cells were kept under normal growth conditions at 80% confluency in six well plates and were transfected with 1 μg of the plasmid harboring Lyn targeting-gRNA and Cas9 expression sequences using Lipofectamine (Lipofectamine 2000 transfection reagent and Opti-MEM reduced serum medium). After 72 h, the successfully transfected cells were selected by culture in puromycin (2 μg/ml) for several days before single cell colonies were generated by serial dilution.

### StromaNKtert cells

Cas9-EGFP-expressing lentivirus (Addgene #63592) was used to generate a constitutive expression of Cas9 in StromaNKtert cells. Therefore, 80% confluent cell layers were transfected in six well plates. Successfully transduced cells were selected by puromycin (2 μg/ml) before single cell colonies were generated by serial dilution. Then, Lyn was knocked out by transfecting those cells with Lyn-crRNA (Dharmacon) in the presence of 1:1 tracrRNA (Dharmacon) using Lipofectamine (Lipofectamine 3000 transfection reagent and Opti-MEM reduced serum medium) according the manufacturer’s instructions. Successful knockout of Lyn in both cell lines was validated by western blotting.

### Generation of CD248-deficient HS-5 cells

CD248-deficient cells were generated using the Gene Knockout kit v2–CD248 (Synthego, Redwood City, CA, USA). To this end, HS-5 wt cells (5 × 104 cells/ml) were incubated in a 24-well plate with serum-free RPMI-1640 medium for 24 h. Next, a ribonucleoprotein (RNP) complex solution (1.3:1 sgRNA to Cas9 ratio) or control (Cas9 only) was mixed with Lipofectamine (CRISPRMAX transfection reagent and Opti-MEM reduced serum medium) and incubated for 10 min at room temperature. Then, the cells were treated with this RNP-transfection solution or the control and cultivated for 3 days. Fresh medium was added after 24 h. After 3 days, the cells were split and grown into single cell clones, maintained for several weeks for further selection and validated by western blotting.

### Western blot

Lysates of HS-5 and StromaNKtert cell variants were made in RIPA lysis buffer (0.607 *g* Tris, 0.876 *g* NaCl, 0.1 *g* SDS, 0.5 *g* sodium deoxycholate, 1 ml Triton X-100 in 49 ml H_2_O). A proteinase inhibitor cocktail mix (Sigma Aldrich, Taufkirchen, Germany, cat# P2714) was added to prevent protein degradation. Before gel loading, protein concentrations were adjusted in the lysates *via* Bradford assay (Sigma). In total, 30 μg of protein was loaded per condition and separated by SDS-PAGE on 12% acrylamide gels or NuPage 4–12% under reducing conditions. Proteins were transferred to a polyvinylidene fluoride (PVDF) membrane (Merck Millipore, Burlington, MA, USA). Membranes were blocked with bovine serum albumin (5% w/v; 1 h; room temperature) in Tris-buffered saline containing 0.1% Tween-20 (TBST), after which membranes were incubated with the primary antibodies at 4°C for overnight incubation. After washing with TBST and subsequent milk block (5% w/v; 1 h), membranes were incubated for 2 h at room temperature with anti-rabbit IRDye 800CW, or anti-mouse IRDye 800CW (both LI-COR and used at 1:10,000). Images were collected using a scanner imaging system (Odyssey CLx LI-COR).

### Mass spectrometry

Extracellular vesicles were generated and purified as described above. The EVs were pelleted by ultracentrifugation for 90 min at 110,000 × *g* and aliquots were lysed in 10 μL of SP3 buffer (5% SDS in PBS). Nucleic acids were degraded in a bioruptor before dithiothreitol (DDT) was added to a final concentration of 5 mM, vortexed and incubated for 30 min at 55°C. Chloroacetamide was added to a final concentration of 40 mM and further incubated for 30 min in the dark and digested with trypsin. After centrifugation at 20,000 × *g* for 10 min the supernatants were transferred into a new tube and adjusted to 1 μg/mL.

All samples were analyzed on a Q Exactive™ Plus Orbitrap (Thermo Scientific, Waltham, MA, USA) mass spectrometer that was coupled to an EASY nLC (Thermo Scientific, Waltham, MA, USA). Peptides were loaded with solvent A (0.1% formic acid in water) onto an in-house packed analytical column (50 cm—75 μm I.D., filled with 2.7 μm Poroshell EC120 C18, Agilent). Peptides were chromatographically separated at a constant flow rate of 250 nl/min using the following gradient: 3–4% solvent B (0.1% formic acid in 80% acetonitrile) within 1.0 min, 4–27% solvent B within 119.0 min, 27–50% solvent B within 19.0 min, 50–95% solvent B within 1.0 min, followed by washing and column equilibration. The mass spectrometer was operated in data-dependent acquisition mode. The MS1 survey scan was acquired from 300 to 1,750 m/z at a resolution of 70,000. The top 10 most abundant peptides were isolated within a 1.8 Th window and subjected to higher-energy collisional dissociation (HCD) fragmentation at a normalized collision energy of 27%. The automatic gain control (AGC) target was set to 5e5 charges, allowing a maximum injection time of 55 ms. Product ions were detected in the Orbitrap at a resolution of 17,500. Precursors were dynamically excluded for 25.0 s.

All mass spectrometric raw data were processed with Maxquant (version 1.5.3.8) using default parameters. Briefly, MS2 spectra were searched against a canonical human Uniprot fasta file (UP000005640, downloaded at: 26.08.2020) database, including a list of common contaminants. False discovery rates (FDR) on protein and peptide spectrum match (PSM) level were estimated by the target-decoy approach to 1% (Protein FDR) and 1% (PSM FDR), respectively. The minimal peptide length was set to seven amino acids and carbamidomethylation at cysteine residues was considered as a fixed modification. Oxidation (M) and Acetyl (Protein N-term) were included as variable modifications for whole proteome searches. The match-between runs option was enabled. Initial data filter was done with Perseus (version 1.6.1.1). The mass spectrometry proteomics data have been deposited to the ProteomeXchange Consortium *via* the PRIDE partner repository with the dataset identifier PXD036932.

### Proteomics

An in-house pipeline combining several R packages [Differential Enrichment Analysis (DEF), limma, clusterProfiler (20–22)] was written and followed. The pipeline includes pre-processing steps, normalization, logarithmic transformation and differentially expressed proteins were identified using limma R package (a combination of a linear model and empirical Bayesian estimation of variance). Potential contaminants and reverse protein sequences were removed along with proteins with too many missing values (only proteins with intensities quantified in three replicates of at least one condition were kept). Normalization was performed using vsn and remaining missing values were classified in two categories as described in the MSnbase R package for further imputation (23). Those resulting from absence of detection of a feature, despite being present at detectable concentrations are expected to be randomly distributed in the data and were handled as missing at random (MAR) and imputed with maximum likelihood-based method (MLE) using the expectation-maximization algorithm. However, biologically relevant missing values resulting from the absence of low abundant ions (below the instrument detection limit) were classified as missing not at random (MNAR) and imputed with a left-censored approach using a deterministic minimal value (MinDet). Information regarding whether a protein normalized intensity value was imputed or not can be found in Supplementary Table 1. Differentially expressed proteins were identified under the following condition (*p*-adjusted ≤ 0.05; 1 ≤ log_2_ fold change ≤ −1). Afterwards, Gene Ontology (GO) and Reactome enrichment analyses of differentially expressed proteins were carried out using clusterProfiler and ReactomePA R packages, respectively (FDR of 5%) (24).

### Statistical analysis

If not stated otherwise, all experiments were performed in at least three independent replicates. Results obtained from representative experiments are shown. Data (*n* ≥ 3) are presented as mean ± standard errors of the mean (SEM). Using the following legend: non-significant (ns) = *p* > 0.05, * = *p* ≤ 0.05, ^**^ = *p* ≤ 0.01, ^***^ = *p* ≤ 0.001, ^***^ = *p* ≤ 0.0001, ^****^ = *p* ≤ 0.0001. Data were analyzed and graphics were constructed using GraphPad Prism v.9.4.1 (GraphPad Software, San Diego, CA, USA).

## Results

### The tyrosine kinase inhibitor Dasatinib reduces the release of EVs from stromal cells

**FIGURE 1:**
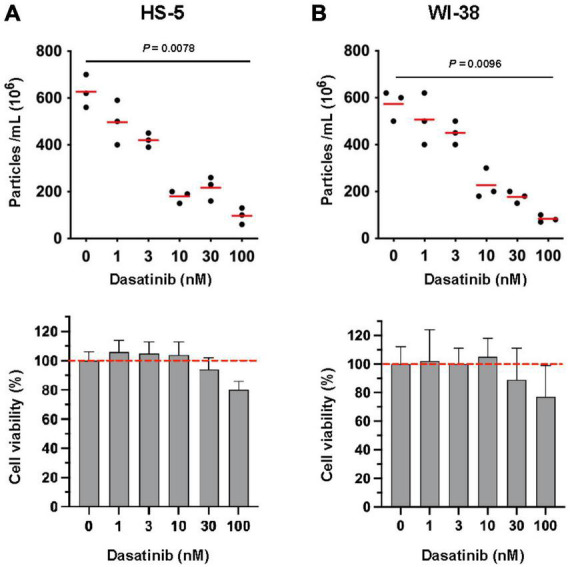
Influence of tyrosine kinase inhibitor Dasatinib on the release of extracellular vesicles (EVs) in stromal cells. HS-5 **(A)** and WI-38 **(B)** cells (2 × 10^6^/ml) were cultivated at 37°C in serum-free RPMI-1640 with dilution series of Dasatinib as indicated. DMSO (0.1% in PBS) served as a control. After 24 h, cells and the supernatant were separated by centrifugation (10 min, 300 × *g*). The supernatant was cleared by centrifugation at 3,000 × *g* and afterwards subjected to ultracentrifugation (90 min, 110,000 × *g*). After ultracentrifugation, the EV pellet was suspended in PBS and the count was determined by nanoparticle tracking analysis. The cell pellet was used for cell viability determination. The viability of the DMSO-treated aliquots served as controls (100%). The statistic evaluation was performed by the Kruskal–Wallis test.

Stromal cells support the viability of primary CLL cells through direct cell contact, implicating SFKs such as Lyn, which is expressed in both tumor and bystander cells in the CLL TME (15). Of note, the prototypic family member c-Src was reported to be able to stimulate both the release of EVs and tumor growth (25). We therefore studied whether also Lyn might influence the EV release in stromal cells. Due to the lack of Lyn-specific inhibitors, we initially tested the efficacy of Dasatinib, a SFK-selective inhibitor also inhibiting Lyn. In this initial experiment, we measured particles that were released after 24 h by nanoparticle tracking analysis. We found that subtoxic concentrations of Dasatinib (≤ 10 nM) reduced the particle release in HS-5 and WI-38 cells in a dose-dependent manner to approximately 29 and 40% of the DMSO-treated control (Figures 1A, B). Higher concentrations further reduced the particle release but also affected cell viability. To us, the data provided initial evidence that Dasatinib reduces the particle release in a dose-dependent manner, suggesting that tyrosine kinases such as Lyn might participate in the EV release from stromal cells.

### Lyn stimulates the EV release in stromal cells

**FIGURE 2:**
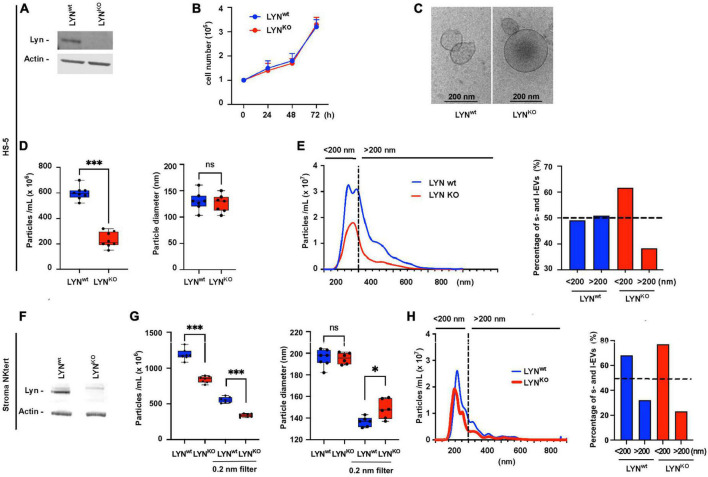
Influence of Lyn on the extracellular vesicles (EV) release in stromal cells. HS-5 cells (wt) and Lyn-deficient counterparts (KO) were compared. **(A)** The expression of Lyn was determined in both cell types by western blot. **(B)** The growth conditions were tested in a kinetic study by determination of the cell count at time points as indicated. **(C)** The EVs in the cell-free supernatants were isolated by sedimentation for 90 min at 110,000 × *g* and visualized by cryo-transmission electron microscopy (TEM). **(D)** To compare the EV concentration both HS-5 cell types were cultivated for 24 h in medium with EV-depleted serum. After precentrifugation steps at 300 × *g*, 3,000 × *g*, and 10,000 × *g*, the EVs were sedimented at 110,000 × *g*, suspended in 500 μL PBS and purified by size exclusion chromatography (SEC). Then, the EV count and diameter of the suspension was determined by nanoparticle tracking analysis. **(E)** To plot which EV size is predominantly influenced by Lyn in HS-5 cells, the EV diameter and count are shown in a histogram. A dashed line separates particles ≤ 200 nm (s-EVs) and ≥ 200 nm (l-EVs). The percentage of particles < 200 nm and > 200 nm is calculated and depicted in a bar chart. The influence of Lyn on the EV release was also tested in the supernatant of wt and Lyn-defective StromaNKtert cells, another stromal cell line. **(F)** The expression of Lyn was determined in both StromaNKtert types by western blot. **(G)** The particle count and diameter were determined by nanoparticle tracking analysis. This time, the EVs were not purified by SEC but aliquots were filtered through a 200 nm filter. **(H)** To plot which EV size is predominantly influenced by Lyn in HS-5 cells, the EV diameter and count are shown in a histogram. A dashed line separates particles ≤ 200 nm (s-EVs) and ≥ 200 nm (l-EVs). The percentage of particles < 200 nm and > 200 nm is calculated and depicted in a bar chart. The results were statistically evaluated by a two-tailed, non-parametric *t*-test (Mann–Whitney) (ns = not significant, *P < 0.05, ^***^P < 0.001).

To directly test the effect of Lyn on the release of EVs, we compared the particle count in the supernatant of Lyn-proficient and Lyn-deficient HS-5 cells. The defect of Lyn in Lyn-deficient cells was confirmed by western blot (Figure 2A). Both variants showed a comparable cell growth and released particles with a typical morphology of EVs, as demonstrated by cryo-transmission electron microscopy (TEM) (Figures 2B, C). We compared the particle diameter and number in the serum-free supernatants of both HS-5 variants (Figure 2D). There was no significant difference in the mean diameter (wt: 129.9 nm and Lyn KO: 125.7 nm, *N* = 8, *P* = 0.8339) but the EV count was significantly reduced in Lyn-deficient cells compared to the wt cells, resulting in a reduction from 595 × 10^6^ to 210 × 10^6^ (*N* = 8, *P* = 0.0002) particles/ml. Comparing the ratio of large (> 200 nm) and small EVs (< 200 nm) in both HS-5 variants, suggests that Lyn appears to affect predominantly but not only the release of larger EVs (Figure 2E). This comparison was also performed with StromaNKtert cells, another stromal cell line. Also here, the Lyn knockdown was confirmed by western blot (Figure 2F). To estimate whether the release of large EVs (l-EVs) or small EVs (s-EVs) is predominantly affected by Lyn, we slightly modified the EV purification procedure. We omitted the 10,000 × *g*-precentrifugation step to avoid the removal of l-EVs. Instead, we filtered aliquots with 200 nm filters to remove l-EVs but left other aliquots untreated before comparing the EV fractions of wt and Lyn KO StromaNKtert. Comparable to HS-5 cells, Lyn-deficient StromaNKtert released less EVs than Lyn-proficient cells (Figure 2G). As expected, filtration caused the removal of larger EVs and reduced the mean diameter from 198/193 to 137/147 nm in Lyn wt and Lyn KO, respectively. However, in both the filtered and unfiltered fractions, Lyn-deficient cells yielded significantly less EVs in comparison to aliquots of wt cells.

Separating the filtered and unfiltered EVs, we found that unfiltered aliquots resulted in a reduction of the mean particle count from 1,189 × 10^6^ in wt cells to 840.4 × 10^6^ in KO cells (*N* = 7, *P* = 0.0006) which is a reduction to 70.6% of the wt control. However, the filtrated aliquots resulted in a reduction of the mean particle count from 553.4 × 10^6^ to 336.6 × 10^6^ (*N* = 7, *P* = 0.0006), which is a reduction to 60.8% of the wt control. These data clearly suggest that the release of both l-EVs and s-EVs from the tested stromal cells is stimulated by Lyn. We also compared the ratio of large (> 200 nm) and small EVs (< 200 nm) in both StromaNKtert variants (Figure 2H). Also in this cell line, Lyn seems to favor the production of l-EVs. However, the effect was much weaker than in HS-5 cells. Therefore, it is not entirely clear if Lyn preferentially affects the release of large vesicles.

### Lyn supports EV uptake in stromal cells

**FIGURE 3:**
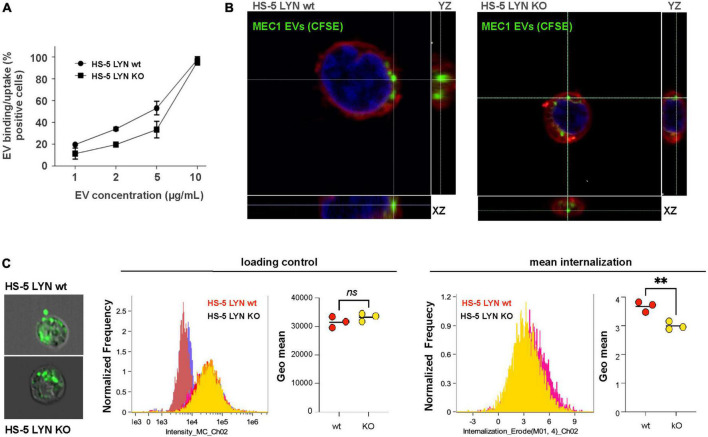
Influence of Lyn from stromal cells on the extracellular vesicles (EV) uptake. **(A)** The uptake/binding of EVs from MEC-1 cells (chronic lymphocytic leukemia, CLL) was tested in Lyn-proficient and Lyn-deficient HS-5 cells. EVs from MEC-1 cells were isolated by ultracentrifugation for 90 min at 110,000 × *g*, stained with DiD and washed with PBS. Both HS-5 cell types were cultivated for 24 h in the presence of a dilution series (1–10 μg/ml) of these EVs. Then, the cells were washed and analyzed by flow cytometry. **(B)** Confocal microscopy of Lyn-proficient and Lyn-deficient HS-5 cells with CSFE-labeled MEC-1 EVs. Cells were incubated with labeled EVs and the cell membrane was subsequently stained with CellMask deep red. **(C)** The internalization process was further analyzed by imaging flow cytometry investigation of 10,000 wt and Lyn-deficient HS-5 cells. Representative images are shown of Lyn-proficient (upper left) and Lyn-deficient HS-5 cells (lower left), incubated with CSFE-labeled EVs for 60 min at 37 °C. The loading controls of three independent experiments with their geo means are shown in the middle panel and the internalized intensity (internalization erode) of the green fluorescence with the geo means is depicted (right panel). The results were statistically evaluated by a two-tailed, parametric *t*-test (Mann–Whitney) (^**^P < 0.01, ns, not significant).

To test the influence of Lyn on the uptake of EVs, we applied different concentrations of MEC-1 EVs to Lyn-proficient and Lyn-deficient HS-5 cells. As demonstrated by flow cytometry, there was a dose-dependent binding to both cell types (Figure 3A). At high EV concentrations (10 μg/ml) almost all cells of both cell types showed EV binding. At lower concentrations (1, 2, and 5 μg/ml), there was a tendency for a better binding to the wt cells. This test cannot discriminate between binding and uptake. Confocal images suggested that both cell types are in principle able to take up EVs (Figure 3B). Imaging flow cytometry was performed to compare the extent of EV uptake between Lyn-proficient and Lyn-deficient HS-5 cells. Therefore, images of 10,000 wt and Lyn-deficient HS-5 cells were evaluated. Figure 3C shows that, despite a comparable loading of target cells with fluorescence-labeled EVs (loading control), the wt HS-5 cells internalized significantly more EVs than the Lyn-deficient counterparts (mean internalization), indicating that Lyn does not only influence the EV release but also contributes to the uptake of EVs by stromal cells.

### Influence of Lyn on filopodia

**FIGURE 4:**
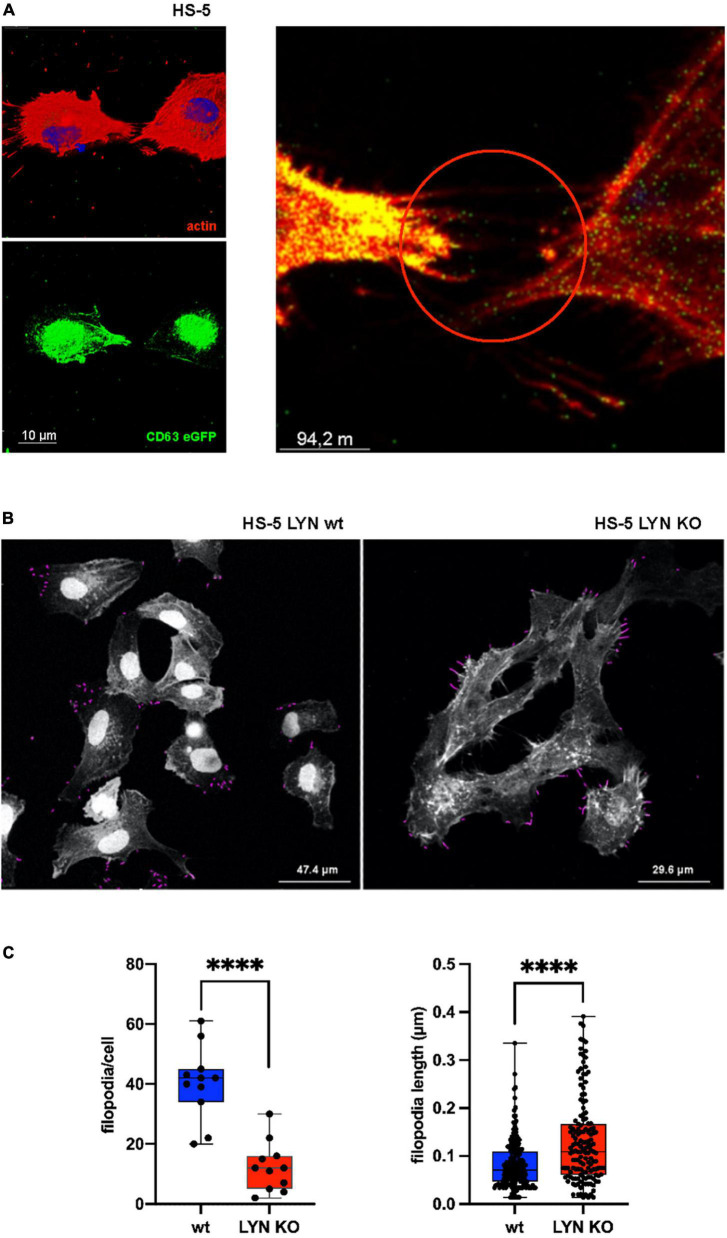
Influence of Lyn on filopodia in stromal cells. **(A)** wt HS-5 cells were transfected with CD63-eGFP and grown to confluency. Then, cells were treated with Alexa Fluor Phalloidin (594) to stain F-actin. Images with single stains and a fused image are depicted. Arrow heads show protrusion-associated CD63^+^ extracellular vesicles (EVs). **(B)** Filopodia were identified in Lyn-proficient and -deficient HS-5 cells after F-actin staining. Filopodia were measured and calculated in fixed cells using the FiloQuant plugin of Fiji software (19). **(C)** Filopodia count per cell and filopodia length were determined. The results were statistically evaluated by a two-tailed, unpaired, non-parametric *t*-test (Mann–Whitney) (^****^P < 0.0001).

Filopodia are dynamic protrusions that oscillate into the cell environment. They enlarge the cell surface, sense and communicate with the extracellular environment and, in addition, bind and guide EVs, as shown with CD63-eGFP-labeled EVs from MEC-1 cells on HS-5 wt cells (Figure 4A) (26, 27). Stromal cells generate protrusions such as filopodia to sense their vicinity and communicate with the local environment. Thus, filopodia might play a decisive role in the EV-based intercellular communication. To address this, we studied the role of Lyn in influencing the count and length of filopodia. Confocal images were prepared of fixed and actin-stained HS-5 wt and HS-5 Lyn-KO cells. As depicted in Figure 4B, images were processed to identify filopodia-like protrusions with the help of the FiloQuant-plugin of the Fiji software (28). Eleven cells were evaluated to calculate the protrusion number, resulting in a significant drop of the mean filopodia number in Lyn-deficient HS-5 cells as compared to wt cells (42 vs. 12 filopodia/cell, *N* = 11 cells, *P* < 0.0001; Figure 4C). Surprisingly, the filopodia from the HS-5 Lyn KO cells were significantly longer (mean of 0.1090 μm, *N* = 146) than those from wt cells (mean of 0.071 μm, *N* = 194). These data suggest that Lyn might be involved in both, the dynamic formation and degradation of filopodia. Further studies are necessary to resolve the diverging effects.

### Lyn supports the generation of CLL-supportive EVs

**FIGURE 5:**
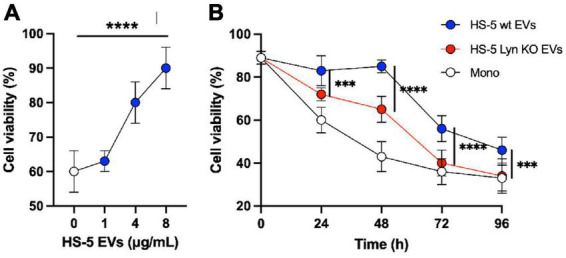
Lyn contributes to generate chronic lymphocytic leukemia (CLL)-supportive extracellular vesicles (EVs). **(A)** Primary CLL cells (0.3 × 10^6/^well in a 96-well plate) of 11 different donors were cultivated in RPMI-1640 medium with 10% FBS and a dilution series of purified EVs from HS-5 cells as indicated. After 24 h the cell viability was determined with CellTiter-Glo 2.0. Statistics: Brown–Forsythe ANOVA test. **(B)** The differential effect of EVs (4 μg/ml) from Lyn-proficient and -deficient HS-5 cells on the viability of primary CLL cells was compared with untreated CLL cells in a kinetic study (0–96 h). At the end of the indicated incubation period, the cell viability was determined with CellTiter-Glo 2.0. Statistics: two-tailed, unpaired *t*-test with Welch’s correction at indicated time points (^***^P < 0.001, ^****^P < 0.0001).

Primary CLL cells rapidly die in *in vitro* cell culture. To test the supportive influence of stromal cell EVs on the viability of CLL cells, we incubated CLL cells of 11 different donors for 24 h with stromal cell EVs at various concentrations (0–8 μg/ml). As shown in Figure 5A, EVs significantly support the cell viability in a dose-dependent manner (*P* < 0.0001) in comparison to the untreated culture of CLL cells (0 μg/ml). We showed already that Lyn stimulates the release of EVs. We now tested if Lyn also influences the quality of supportive EVs. Therefore, in a kinetic study, we cultivated primary CLL cells with 4 μg EVs from Lyn-proficient, Lyn-deficient HS-5 cells or without addition of vesicles as a control (Figure 5B). Under these conditions, both EV types supported the viability of CLL cells as compared to aliquots without EV treatment (Mono). However, the EVs from wt HS-5 cells supported the viability of CLL cells significantly stronger than EVs from Lyn-deficient cells. This was also shown by the area under the curve (AUC) which was calculated as follows: 6,996, 5,724, and 4,800 with wt EVs, EVs from Lyn-defective HS-5 cells and without EVs, respectively. After 48 h, the supportive effect of EVs from both cell types decreased. Notably, in EVs from Lyn KO cells, the support is reduced to the monoculture (background) level after 96 h of coculture. Since we applied the same EV concentration in the wt and knockout setting, the results suggest that not only the EV count but also the quality of the EVs is influenced by Lyn.

### Lyn influences the protein composition of EVs from HS-5 cells

**FIGURE 6:**
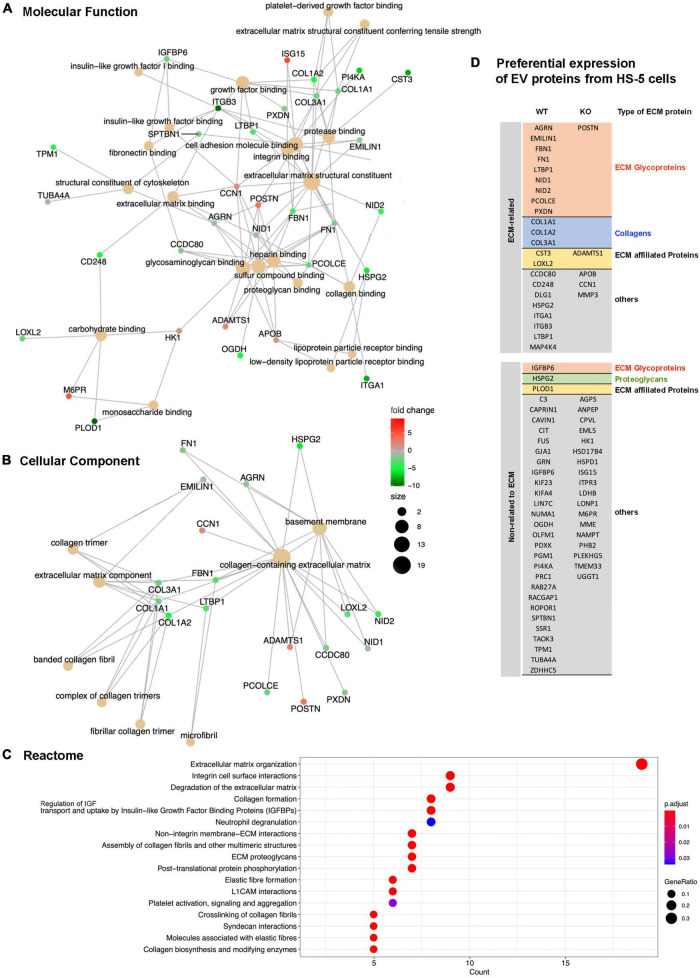
Proteomic study of extracellular vesicles (EV) proteins from Lyn-proficient and deficient HS-5 cells. Differentially expressed proteins from EVs of Lyn-proficient and -deficient HS-5 cells were identified under the following condition (*p*-adjusted ≤ 0.05; 1 ≤ log_2_ fold change ≤ –1) (Supplementary Table 1). Gene Ontology (GO) studies are depicted, i.e., molecular function **(A)**, cellular component **(B)**, and the Reactome **(C)**. Enrichment of proteins with a relation to the extracellular matrix are shown **(D)**.

To address differences in the protein composition of EVs from Lyn-proficient and deficient HS-5 cells we employed mass spectrometry. A total of 70 proteins were FDR significant and evaluated in a proteomic study (Supplementary Table 1). GO enrichment analysis was performed of these hits. The following aspects were studied: Molecular function (Figure 6A), cellular component (Figure 6B), Reactome (Figure 6C). An analysis of the biological processes (BP) was also performed (Supplementary Table 1). All studies showed an enrichment of proteins related to the extracellular matrix. This includes terms such as extracellular structure or matrix organization or collagen-containing extracellular matrix. This tendency was more pronounced in EVs from Lyn wt cells as compared to the EVs from Lyn-deficient cells. This includes an overrepresentation of proteins related to the ECM including fibronectin (FN), collagen (Col1A1/2 and Col3A1), CD248, integrins (ITGA1, ITGB3), Elastin Microfibril Interfacer 1 (EMILIN1), Fibrillin 1 (FBN1), Fibronectin (FN1), among others (Figure 6D and Supplementary Table 1). Most of them were ECM categorized as glycoproteins. These findings suggest that the EVs, particularly those generated by cells expressing Lyn, may play a role in determining the ECM composition, structure and function.

### CD248 contributes to the support of CLL cells

**FIGURE 7:**
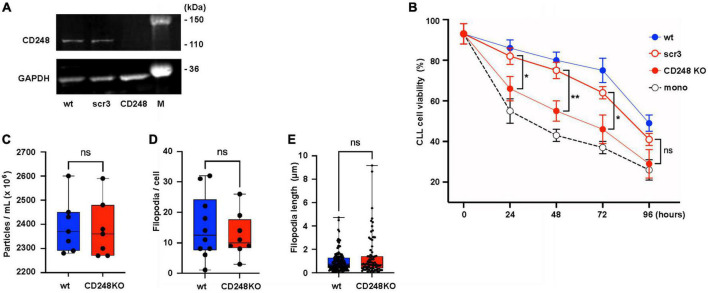
CD248 contributes to the support of chronic lymphocytic leukemia (CLL) cells. **(A)** CD248-defective HS-5 cells were generated. The defect was confirmed by western blot in cell lysates of HS-5 wt, CD248 knockout (CD248 KO), and the scrambled control (scr3). **(B)** Primary CLL cells (1.5 × 10^6^/ml) were cocultivated with HS-5 cell variants as indicated (1 × 10^4^/ml) or without HS-5 cells. After indicated time points, the non-adherent CLL cells were suspended and transferred to another well for the cell viability measurement using CellTiter-Glo2 as indicated. The results show the means ± SEM of nine different patients. Statistics is shown between CD248 KO and the scr3 control at indicated time points. **(C)** To test the influence of CD248 on the release of particles, wt and CD248-defective HS-5 cells were cultivated for 24 h in medium with extracellular vesicles (EV)-depleted serum. Then, the particles count was determined in the supernatants of seven aliquots by nanoparticle tracking analysis. **(D,E)** Filopodia were identified in fixed and F-actin-stained confocal images of wt and CD248 KO HS-5 cells. The filopodia count per cell and filopodia length were measured and calculated using the FiloQuant plugin of Fiji software (19). The non-parametric, two-tailed *t*-test (Mann–Whitney) was performed when indicated (*P < 0.05, **P < 0.01, ns, not significant).

We found that EVs from Lyn-deficient HS-5 cells supported the survival of primary CLL cells less than EVs from wt HS-5 cells. Looking for proteins which might be responsible for this diverging efficacy, we found that the EVs from wt HS-5 cells had the tendency to express more proteins with a connection to the extracellular matrix (Figure 6D). One of the candidate proteins is CD248. To test whether CD248, a down-stream target of Lyn, might at least in part account for the CLL support in cocultures of primary CLL and stromal cells, we generated a knock-down of CD248 in HS-5 cells (Figure 7A). In coculture experiments of primary CLL cells without, with HS-5 cells, with HS-5 CD248 knock-down or scrambled control cells (scr3), we determined the viability of the CLL cell aliquots at different time points for up to 96 h (Figure 7B). A direct comparison of the CLL cell viability in HS-5 CD248 KO or HS-5 Src control cocultures revealed a reduced support by CD248 KO cells at all tested time points (24, 48, 72, and 96 h). Except for the 96 h-time point, the reduction was statistically significant, i.e., *P* = 0.0436, *P* = 0.0069, *P* = 0.0379, and *P* = 0.1438 after 24, 48, 72, and 96 h, respectively. To show the overall influence of the HS-5 variants on CLL cell survival we determined the AUC as follows: wt HS-5 cells (7,488), scr control (6,912), CD248-deficient HS-5 cells (5,472), and the CLL monoculture (4,668) confirming that CD248 from HS-5 cells contributes at least in part to the survival support of primary CLL cells. In contrast to Lyn (Figure 4), CD248 showed no significant influence on the particle release (Figure 7C), the filopodia count (Figure 7D) and the filopodia length (Figure 7E).

## Discussion

To appreciate the role of stromal cell EVs in supporting malignant CLL cells, we first need to highlight the function of the stromal cells. The malignant B cells in CLL undergo spontaneous apoptosis if not effectively stimulated through multiple interactions with supporter cells within the lymphoid tissue, the so-called TME. Follicular dendritic cells and mesenchymal stromal cells substantially contribute to this stimulation. In cooperation with endothelial cells, they are involved in CLL cell homing (29). Additionally, they are influenced by the tumor cells themselves to develop an improved supportive phenotype, the so-called CAF phenotype. The survival signaling is mainly facilitated by cell contact, with multiple interactions, including adhesion molecules, receptor-ligand interactions and soluble growth factors (7). EVs also contribute to the mutual activation. In fact, CLL patients have an increased amount of EVs with a CLL signature in the peripheral blood (14). CLL cell-derived EVs are able to induce a CAF-like phenotype in stromal cells (30). The EVs from CLL cells show a mRNA enrichment for kinases of the BCR pathway and the EV release might be influenced by kinases of the BCR pathway, since treatment with inhibitors of BTK and PI3K reduces the plasma EV count in CLL patients. This effect was not shown with fludarabine, which does not target kinases (14). Because Lyn (i) is an upstream non-receptor tyrosine-protein kinase within the BCR signaling and other receptor signaling pathways, it is (ii) highly expressed in CLL and bystander cells of the B-CLL TME (15, 31), and (iii) inhibition of Lyn downstream kinases BTK and PI3K reduces the EV release, we speculated that Lyn might also influence the tumor-supportive EV communication of stromal cells, both by raising the EV release (quantity) and influencing the EV composition (quality).

The main findings of this paper are that subtoxic tyrosine kinase inhibition and the knockout of Lyn in stromal cell lines significantly reduces the EV release and the EV uptake as compared to the untreated or Lyn-proficient counterparts (quantity). Secondly, Lyn helps to generate CLL-supportive EVs. Lyn modifies the protein composition of EVs with a tendency to produce proteins that are associated to the extracellular matrix and interacting cell membrane molecules, such as integrins, collagen, fibronectin and CD248. As an example, the depletion of CD248 in stromal cells resulted in a reduced survival support of primary CLL cells as compared to the Lyn-proficient stromal cells (quality).

### Lyn raises the EV quantity

Lyn stimulates not only a survival cascade, implicating BTK, PI3K and other downstream kinases, but also stimulates the formation of protrusions by phosphorylating HS1 (hematopoietic cell-specific Lyn substrate 1), which then facilitates the formation of the Arp2/3 complex and starts the F-actin nucleation process, a prerequisite for the formation of protruding filopodia (18). Filopodia protrude transiently into the extracellular space and function as antennae to sense the cell environment (32, 33). Filopodia retraction requires actin degradation. Because actin is a major hit in EV proteomics and we speculated that interstitial shear forces might pinch parts of the filopodia to form EVs. Indeed, Lyn inhibition or depletion resulted in a reduced release of EVs supporting our hypothesis (Figures 1–3). However, the role of Lyn might be more complex, because we also observed a reduction of smaller EVs which generally derive from multivesicular endosomes and not from the cell membrane indicating that Lyn also influences the release of s-EVs. Also, the EV uptake was reduced in Lyn-deficient cells, which correlates with a reduced number of filopodia per cell. Taken together, Lyn increases the amount of released EVs, however the exact mechanisms how Lyn influences the EV release and uptake at the same time, and its influence on filopodia formation and retraction are not fully resolved and deserve further studies.

### Lyn influences the EV quality

GO comparison of the EV proteins from Lyn-proficient and deficient stromal cells (HS-5) showed a depletion of proteins with a link to the extracellular matrix in the absence of Lyn. The overrepresentation of proteins in wt cells with a relation to the ECM included fibronectin (FN), collagen (Col1A1/2 and Col3A1), CD248, integrins (ITGA1, ITGB3), Elastin Microfibril Interfacer 1 (EMILIN1), Fibrillin 1 (FBN1), Fibronectin (FN1), among others. Currently it is an open question if such proteins originally derive from EVs or if they in part, as soluble molecules, stably bind to EVs and form the so-called EV corona (4). CD248 (endosialin, tumor endothelial marker 1) is a membrane-spanning glycoprotein, which along with thrombomodulin belongs to the C-type lectin-like receptor family (10, 34). In addition to pericytes and CAFs, it is expressed in malignant cells of a few solid tumors. CD248 is restricted to the cell membrane and EVs and able to bind collagen and fibronectin. Thus, it links the plasma membrane with the ECM. Because it is also released in EVs, it might even link structural elements of the ECM beyond the cell membrane. CAF produce an extracellular matrix with a higher density as compared to normal fibroblasts and a high ECM density is an important driver of the tumor growth (35). Thus, mechanisms that contribute to ECM stiffness might also contribute to the interaction between tumor and bystander cells. Also, in our experiments, we were able to show that in a co-culture of primary CLL cells and HS-5 fibroblasts, the wt fibroblasts produced a significant better CLL support than the CD248 KO HS-5 cells (Figure 7). This was an initial experiment to demonstrate that the ECM plays an important role in the crosstalk between tumor and bystander cells. Experiments with a deletion of other ECM hits (see above) are in preparation.

The functionality of CAFs and in particularly EVs from such stromal cells are appreciated as important drivers for the survival of B-CLL cells in the TME of CLL-affected lymphoid tissue. Such EVs prolong the survival of primary CLL cells and generate chemoresistance to standard treatment options in CLL, such as Cladribin, Idelalisib, Venetoclax, Ibrutinib, and Bortezomib (5, 36). Much alike a direct contact between CLL and stromal cells, which is an established survival signal for CLL cells, the EVs are also able to present multiple supporting molecules in a membrane-associated context. In addition, if EVs are taken up, they will deliver proteins and nucleic acids to the target cell. However, the impact of such mediators is dose-dependent and fades with the distance between donor and recipient cells (Figure 5). To overcome this dilution in tissue many cells including stromal cells, extend their surface by forming protrusions, which allows cell contact over distance. Using associated proteinases to partially cleave the ECM, such filopodia were shown to directly guide EVs to the distant target cells (27). Thus, the composition and the arrangement of the structural elements of the ECM might play a decisive role in the tumor-supporting cross-talk between tumor and supporting stromal cells.

## Data availability statement

The datasets presented in this study can be found in online repositories. The names of the repository/repositories and accession number(s) can be found below: https://www.ebi.ac.uk/pride/archive/, PXD036932.

## Ethics statement

The studies involving human participants were reviewed and approved by the Geschäftsstelle der Ethikkommission, Universität zu Köln. The patients/participants provided their written informed consent to participate in this study.

## Author contributions

TO was the main investigator who performed most experiments. AS generated Lyn-deficient HS-5 and StromaNKtert cells. RR-R performed the proteomics. LL performed the western blots and several confocal images. ML and OJ performed the ImageStream analyses. PW performed and processed the mass spectrometry. P-HN and MH helped with valuable input and manuscript writing. HH designed, evaluated, and supervised the project and wrote the manuscript. All authors read and approved the final version of the manuscript.

## Supplementary material

Supplementary Table 1 Lists of differentially expressed proteins, which were identified under the following condition (*p*-adjusted ≤ 0.05; 1 ≤ log_2_ fold change ≤ −1). Lists of Gene Ontology (GO) and Reactome enrichment analyses of differentially expressed proteins were carried out using clusterProfiler and ReactomePA R packages, respectively (false discovery rate of 5%).
